# Rapid Room-Temperature Synthesis of Mesoporous TiO_2_ Sub-Microspheres and Their Enhanced Light Harvesting in Dye-Sensitized Solar Cells

**DOI:** 10.3390/nano10030413

**Published:** 2020-02-27

**Authors:** Mohammad Alduraibi, Mahmoud Hezam, Bader Al-Ruhaimi, Ahmed Mohamed El-Toni, Ahmad Algarni, M. Abdel-Rahman, Wang Qing, Abdullah Aldwayyan

**Affiliations:** 1 Physics and Astronomy Department, College of Science, King Saud University, Riyadh 11451, Saudi Arabia; albader2030@gmail.com (B.A.-R.); 438105503@student.ksu.edu.sa (A.A.);; 2 King Abdullah Institute for Nanotechnology, King Saud University, Riyadh 11451, Saudi Arabia; aamohammad@ksu.edu.sa; 3 Central Metallurgical Research and Development Institute, CMRDI, Helwan 11421, Cairo, Egypt; 4 National Center for Nanotechnology and Semiconductors, King Abdulaziz City for Science and Technology (KACST), Riyadh 11421, Saudi Arabia; 5 Electrical Engineering Department, College of Engineering, King Saud University, Riyadh 11421, Saudi Arabia; mabdelrahman@ksu.edu.sa; 6 Department of Materials Science and Engineering, National University of Singapore, Singapore 117576, Singapore; qing.wang@nus.edu.sg

**Keywords:** titanium dioxide, dye-sensitized solar cells, anatase, surfactant, CTAB, light scattering

## Abstract

Submicron sized mesoporous spheres of TiO_2_ have been a potential alternative to overcome the light scattering limitations of TiO_2_ nanoparticles in dye-sensitized solar cells (DSSCs). Currently available methods for the growth of mesoporous TiO_2_ sub-microspheres involve long and relatively high temperature multi-stage protocols. In this work, TiO_2_ mesoporous sub-microspheres composed of ~5 nm anatase nanocrystallites were successfully synthesized using a rapid one-pot room-temperature CTAB-based solvothermal synthesis. X-Ray Diffraction (XRD) showed that the grown structures have pure anatase phase. Transmission electron microscopy (TEM) revealed that by reducing the surfactant/precursor concentration ratio, the morphology could be tuned from monodispersed nanoparticles into sub-micron sized mesoporous beads with controllable sizes (50–200 nm) and with good monodispersity as well. The growth mechanism is explained in terms of the competition between homogeneous nucleation/growth events versus surface energy induced agglomeration in a non-micelle CTAB-based soft templating environment. Further, dye-sensitized solar cells (DSSCs) were fabricated using the synthesized samples and characterized for their current-voltage characteristics. Interestingly, the DSSC prepared with 200 nm TiO_2_ sub-microspheres, with reduced surface area, has shown close efficiency (5.65%) to that of DSSC based on monodispersed 20 nm nanoparticles (5.79%). The results show that light scattering caused by the agglomerated sub-micron spheres could compensate for the larger surface areas provided by monodispersed nanoparticles.

## 1. Introduction

Dye-sensitized solar cells (DSSCs), since invented by O’Regan and Gratzel in 1991 [1], have been a promising low-cost photovoltaic technology [2,3,4,5,6,7]. In a typical DSSC, solar energy photons are absorbed by compliantly absorbing dye molecules that are loaded on a mesoporous film of TiO_2_ nanoparticles (typically ~20–30 nm). The dye-photogenerated electrons are subsequently injected into the TiO_2_ nanoparticles, through which they travel to the device photoanode. The TiO_2_ nanoparticles work, therefore, as an electron acceptor as well as an electron transport medium. The high surface area associated with the morphology of TiO_2_ nanoparticles plays a vital role in determining the amount of dye loading and therefore the amount of generated electrons. The morphology of TiO_2_ nanoparticles also determines the transport path which photoelectrons will subsequently take in their journey towards the external circuit through the thick layer of TiO_2_ nanoparticles [8,9,10].

Besides the effects on dye-loading and electron transport mentioned above, the morphology of TiO_2_ nanoparticles can have beneficial scattering effects on the incident solar light [11,12,13,14,15,16,17,18,19,20,21,22,23,24,25,26,27,28,29]. TiO_2_ films made of nanoparticles with sizes ~20–30 nm usually exhibit high transparency and weak scattering effects. Because of that, a scattering layer of sub-micron sized TiO_2_ structures can be beneficially added to the device structure in order to reflect non-absorbed light back into the dye-loaded TiO_2_ nanoparticles layer. Different morphologies of TiO_2_ sub-micrometer sized structures have been invented for this purpose, e.g., particles [11,12,13,14,15], voids [16,17], and inverse-opal photonic crystals [18,19,20,21]. Due the remarkably smaller surface area accessible in the scattering layer, it can only negligibly contribute to the dye adsorption process. An elegant improvement idea has been to alternatively deposit a TiO_2_ layer made of mesoporous sub-micron beads, resulting in a bi-functional TiO_2_ layer that efficiently works both as a light scatterer and a dye loader [22,23,24,25,26,27,28,29]. The literature on such bi-functional scattering layers mostly report them as an additional layer on top of the nanocrystalline TiO_2_ thin film [22,23,24,25]. Dehong Chen et al., however, reported the use of a single layer of sub-micron sized mesoporous beads that was completely responsible for both dye loading and light scattering [27,28,29]. The work of Dehong Chen et al. continued to attract further research efforts on optimizing the implementation of mesoporous TiO_2_ microspheres in DSSCs [30,31,32].

The method of Dehong Chen et al. involves an initial relatively long sol-gel step followed by a relatively high temperature (~160–200 °C) solvothermal step [27,28,29,30,31,32]. Following the work of Dehong Chen et al., several synthesis methods were developed by other research groups to simplify the synthesis of mesoporous TiO_2_ sub-microspheres. Daesub Hwang et al. used electrostatic spray technique to directly agglomerate the commercially available P25 TiO_2_ nanoparticles into larger mesoporous submicron beads [33]. Dapeng Wu et al. similarly started with P25 nanoparticles in order to synthesize TiO_2_ microspheres that are composed of anatase nanospindles through a multi-step heating approach [34]. Hong-En Wang et al. replaced the solvothermal step by a shorter microwave heating process [35]. Yong Liu et al. reported the successful synthesis of strikingly radially oriented mesoporous TiO_2_ microspheres using a long but relatively low-temperature evaporation-driven assembly method [36]. Zhao-Qian Li et al. could effectively eliminate the first sol-gel step by using different alcoholic solvents [37,38] performing the solvothermal reaction step at 200 °C.

This work reports a novel method for a rapid room-temperature synthesis of anatase TiO_2_ nanocrystals (~5–20 nm) that can controllably agglomerate into bigger sub-micron sized beads (50–200 nm) with good control over their size. The samples are implemented in DSSCs, where light scattering in the agglomerated nanospheres is shown to play an important role for light harvesting. Literally, the smaller surface area available for dye-loading in bigger agglomerates could be compensated by their enhanced light scattering.

## 2. Materials and Methods

All chemicals used in the synthesis were of analytical grade and purchased from SigmaAldrich. They were used without any further purification. First, 6 mM of Hexadecyltrimethylammonium bromide (CTAB) solution was prepared in a mixture of ethanol and DI-water, volume ratio 3:5, and stirred for 30 min. During the stirring process of CTAB solution, the precursor solution was separately prepared by dissolving Tetrabutyl titanate (TBO) in ethanol with concentrations of 60, 120, and 600 µM for samples S1, S2, S3, respectively. The volume ratio between the CTAB solution and the TBO solution was kept at 7:1 for all three samples. The TBO solution was drop-wisely added to the CTAB solution at a rate of ~1 mL/min, and the whole mixed solution was vigorously stirred for two hours. All above steps were carried out under ambient conditions of room temperature and pressure. The resulting solution containing the TiO_2_ samples was filtered and washed thoroughly with de-ionized water and ethanol and dried under vacuum at 60 °C.

Transmission Electron Microscopy (TEM) and high resolution TEM (HRTEM) measurements were carried out using JEOL JEM-2100F HRTEM operated at 200 kV. Surface area measurements were performed using Quantachrome NOVA 4200e Surface Area & Pore Size Analyzer. All samples were analysed using N_2_ adsorption–desorption isotherms at 77 K. The as-grown samples were first heat-treated using the same heating steps used to prepare the DSSC electrodes (mentioned below) in order to maintain conditions that are similar to those in the working device. Before taking the adsorption–desorption isotherms, the samples were further degassed at 200 °C for 3 h in order to remove any adsorbed vapor. The surface area was calculated from the adsorption isotherms using the Brunauer-Emmett-Teller (BET) method. The pore size distribution was calculated by analysing the desorption isotherms using the Barrett-Joyner-Halanda (BJH) method. XRD characterization was carried out using PANalytical X’Pert PRO MPD X-Ray Diffractometer, with Cu-Kα as the X-ray radiation source (λ = 0.154 nm), and a rating of 40 kV, 15 mA in the θ/2θ mode.

The synthesized TiO_2_ samples were used to prepare three different TiO_2_ pastes using a previously reported paste fabrication procedure [3,8]. FTO glasses were cleaned ultrasonically for 30 min by a special detergent solution, then by de-ionized water, and finally by ethanol. The FTO glasses were then placed under hot air stream at 400–500 °C for about 30 min. The prepared pastes were then coated on the cleaned FTO glasses by screen printing using 90 T polyester mesh to print circle-shaped films of 0.28 cm^2^ area. After that, they were dried at 125 °C for 2–3 min. This process was repeated three times to obtain an overall thickness of ~16 µm for all samples. The TiO_2_ films were then sintered at 325 °C for 5 min, 375 °C for 5 min, 450 °C for 15 min, and finally at 500 °C for 15 min. The samples were then soaked in 40 mM TiCl_4_ solution (prepared by dilution of a stock solution that was prepared at 0 °C with 2 M concentration) at 70 °C for 30 min. Then, they were rinsed with water and ethanol and again heated at 500 °C for 15 min. When cooling down to 80 °C, the samples were immersed in N719 dye solution (0.5 mM, in 1:1 volume ratio of acetonitrile and tert-butyl alcohol) and kept there for around 20 h. The counter electrodes were drilled and cleaned by the cleaning process mentioned above. Few drops of H_2_PtCl_6_ solution (2 mg of Pt in 1 mL ethanol) were casted on the cleaned FTO glasses, and then heated at 400 °C to make the Pt film. The cell was fabricated after that by using a thermoplastic spacer between the two electrodes and hot pressing to perform the sealing process. The iodine-based electrolyte solution (0.03 M I_2_, 0.6 M BMII and 0.1 M guanidinium thiocyanate in a mixture of acetonitrile and valeronitrile solvents (volume ratio 85:15)) was introduced between the two electrodes through the drilled hole, which was sealed after that with a thin covering glass.

A Newport class AAA solar simulator was used to provide AM 1.5 G illumination, and light intensity was measured using a calibrated Si reference cell. A Keithley source meter was used for the *I*-*V* measurements under the simulated solar illumination, with a *V_oc_*-*I_sc_* potential direction and a scan rate of 16 mV/s. Incident Photon-to-Current Conversion Efficiency (IPCE) was measured using a 300 W xenon lamp and a spectrometer with 5 nm resolution. The incident photon flux was measured using a calibrated Si photodiode, whereas the collected current was measured using a current amplifier, and both spectrometer and current amplifier were controlled using Newport TRACQ Basic software.

## 3. Results and Discussion

**Figure 1:**
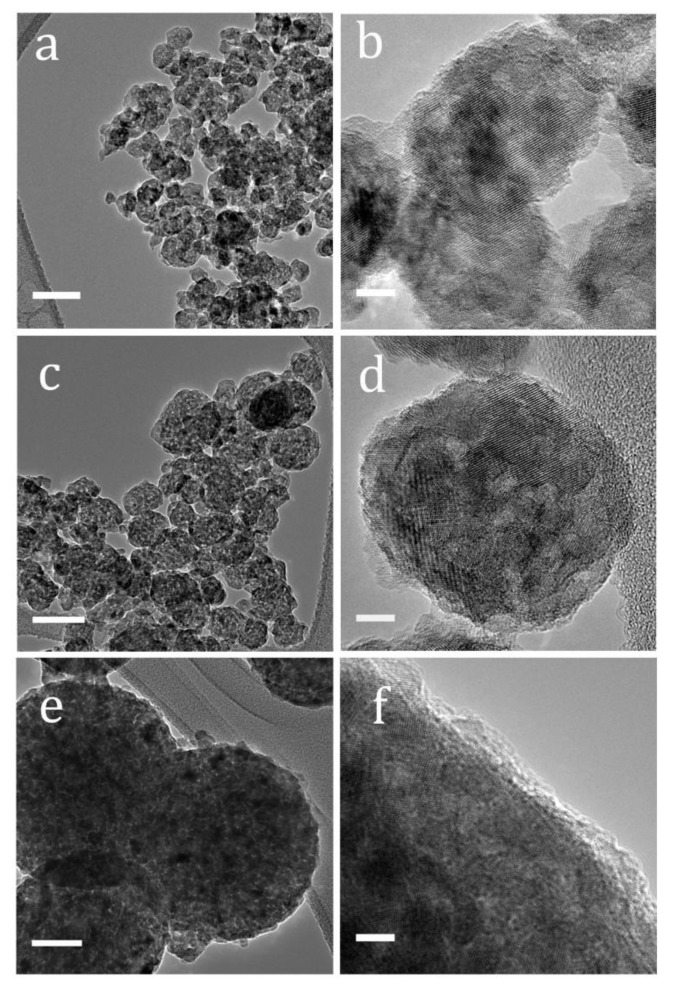
TEM and HRTEM images of sample (**a**,**b**) S1 (**c**,**d**) S2 (**e**,**f**) S3. The scale bars in the TEM images (**a**,**c**,**e**) correspond to 50 nm while the scale bars in the HRTEM images (**b**,**d**,**f**) correspond to 5 nm.

Figure 1 shows the TEM and HRTEM images of the prepared samples. For the S1 sample, when the surfactant/precursor concentration ratio was the highest, weakly agglomerated nanoparticles with sizes between ~15–20 nm were produced (Figure 1a,b). For sample S2, when the surfactant/precursor concentration ratio was reduced, smaller TiO_2_ nanocrystallites (with a crystallite size of ~5 nm, Figure 1d) agglomerated into bigger raspberry-like nanoparticles with sizes of ~50 nm, as can be seen in Figure 1c. For sample S3, with the lowest surfactant/precursor concentration ratio, similarly small (~5 nm) crystallites agglomerated into now bigger sub-microspheres with diameters of around 200 nm (Figure 1e,f).

The growth mechanism can be explained in terms of the CTAB and TBO concentrations as the two main parameters affecting the crystallite size and agglomeration degree in the prepared samples. Surfactants are polar molecules that can electrostatically “template” the growth solution medium. One of these surfactants is the cationic surfactant CTAB which has been used for a long time for such soft templating purpose in the preparation of various porous nanomaterials [39]. During the growth process of TiO_2_ nanocrystallites, precursor ions will be somehow affected by the existence of the polarized surfactant molecules in the solution. The longitudinal CTAB molecules can agglomerate in different shapes, e.g., micelles, based on their concentration. The critical concentration after which the surfactant molecules start forming micelles is called critical micelle concentration (CMC), which is around 0.9 mM for CTAB in pure DI-water [40,41]. Adding ethanol to DI-water increases the CMC value due to the hydrophobic tails of CTAB which interact stronger with ethanol than with pure DI-water [40,41,42]. For 45.3% volume fraction of ethanol in a mixture of ethanol/DI-water, which is the case in this study, CMC can be around 20 mM [41] whereas the overall concentration of CTAB in the reaction mixture is only 5.25 mM for all samples here, which is below the CMC value. Accordingly, we can conclude that there was no formation of CTAB micelles in the growth solutions of all samples.

The growth starts with homogenous nucleation events, whose number and growth rate depend mainly on the initial concentration of the TBO precursor. Therefore, with the lowest TBO concentration used for the preparation of sample S1, it can be assumed that a smaller number of nucleation events occurred during the reaction with slower growth rate and stronger effect of the polar CTAB molecules. This can explain the growth of monodispersed TiO_2_ nanocrystals with ~15–20 nm crystallite size.

With higher TBO concentrations, which is the case for S2 and S3 samples, more homogenous nucleation events are allowed, and these events become spatially closer as well. Adjacent grown nanoparticles are, therefore, more likely to agglomerate, driven by their high surface energy and spatial proximity, before they can grow bigger. This is indeed what was noticed: larger monodispersed nanocrystals for S1 and smaller agglomerated nanocrystals for S2 and S3. The crystallite sizes for S2 and S3 samples were around ~5 nm (as can be seen in the HRTEM images, Figure 1d,f). For S3, the agglomeration effect becomes larger resulting in larger TiO_2_ agglomerates of around 200 nm as shown in Figure 1e.

**Figure 2:**
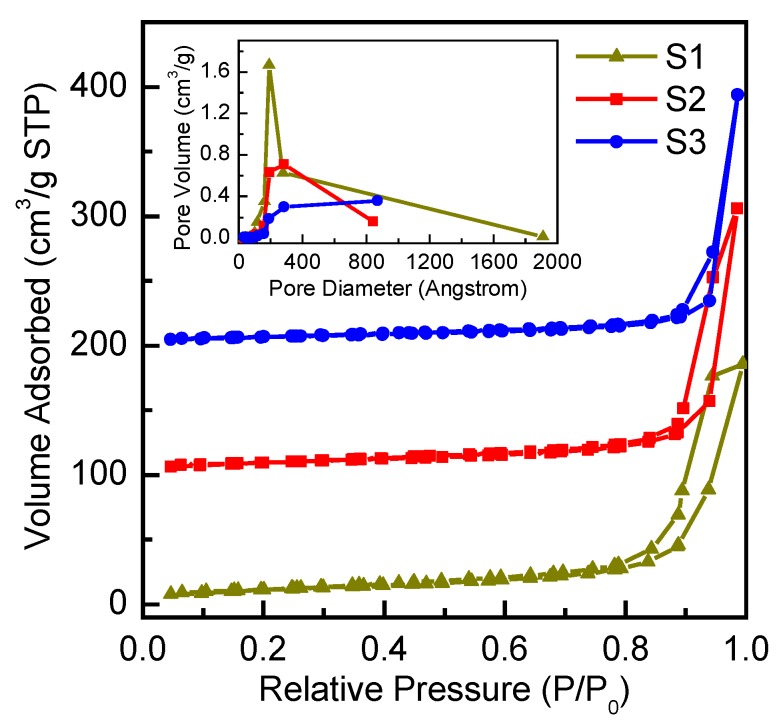
N_2_ adsorption–desorption isotherms for the three prepared samples. The scale in the y-axis corresponds to the S1 sample while, for clarity purposes, the S2 and S3 isotherms are scaled up by 100 cm^3^/g and 200 cm^3^/g, respectively. The inset shows the pore size distributions for the three samples.

Figure 2 shows the N_2_ adsorption/desorption isotherms for the three samples along with the pore size distributions in the inset. All samples exhibit Type IV isotherms with hysteresis loops of type H4 according to the IUPAC classification, a behaviour that is often found in agglomerated nanocrystals [43]. Table 1 shows the calculated textural properties of the three samples. Interestingly, sample S3 has around 40% less surface area compared to that of sample S1. Nevertheless, as will be discussed below, the retrieved photocurrents in the two devices were almost the same.

**Figure 3:**
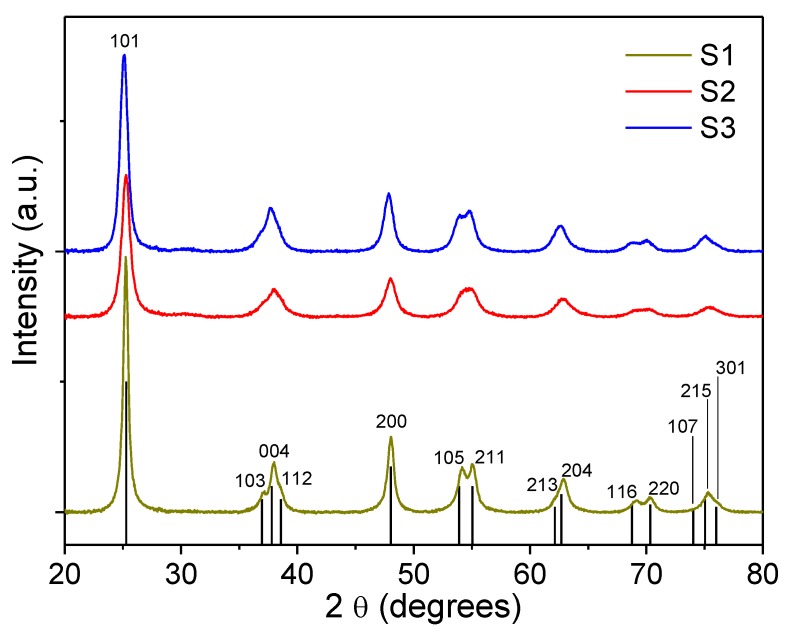
XRD patterns of samples S1, S2, and S3. All samples could be indexed with the JCPDS file No. 21-1272 for anatase TiO_2_ structure, which is presented as black vertical bars at the bottom of the figure.

XRD patterns (Figure 3) for all the powder samples could be indexed to the pure anatase TiO_2_ phase (JCPDS file No. 21-1272). The Scherrer formula was used to estimate the grain size of nanoparticles, and the crystallite sizes were estimated to be 20, 11, and 13 nm for the S1, S2, and S3 samples, respectively. Although overestimated compared to TEM images, the XRD results are broadly in agreement with TEM images: S1 sample showed almost doubled grain size of S2 and S3 samples.

**Figure 4:**
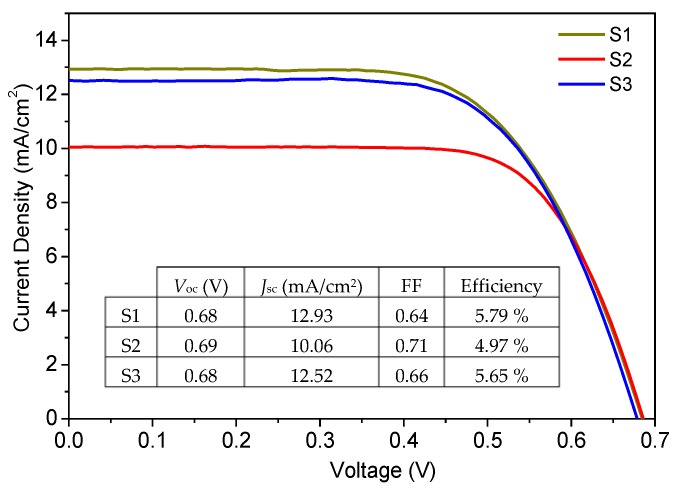
*J*-*V* curves for best devices fabricated using S1, S2, and S3 samples. The strong scattering effect within the TiO_2_ layer in the S3 sample could almost retrieve the photocurrent of S1 made of monodispersed TiO_2_ nanoparticles.

**Figure 5:**
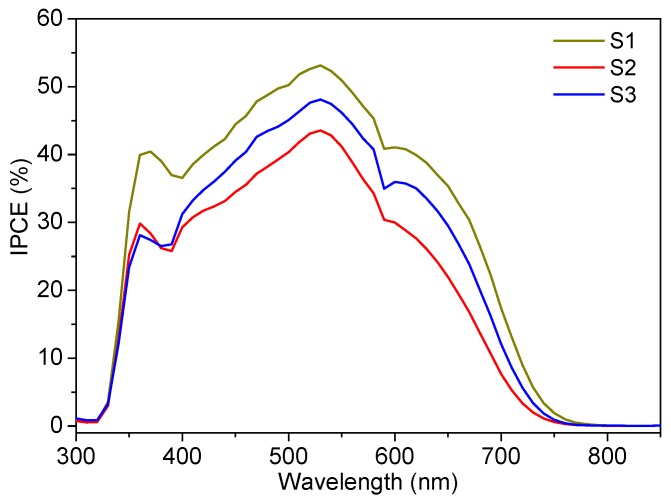
Incident Photon-to-Current Conversion Efficiency (IPCE) spectra for DSSCs fabricated using S1, S2, and S3 samples.

Figure 4 shows the *J*-*V* curves of the best DSSC cells made with each sample. A summary of averaged device characteristics is displayed in Table 2. Open circuit voltage (*V_oc_*) values did not appreciably change for all three samples. However, a clear variation in the short circuit current density (*J_sc_*) values was observed, which are similarly reflected in the IPCE measurements in Figure 5. The current is highest for sample S1 that has monodispersed nanoparticles of 15–20 nm size. This can be due to the higher surface area of sample S1 resulting in higher dye loading and so higher photocurrent. For sample S2, *J_sc_* is reduced most probably due to the reduced surface area in the sample. Despite a smaller crystallite size compared to S1 (bringing about access to more surface area), the agglomeration effect in sample S2 was evidently dominant resulting in ~22% reduction in photocurrent. Sample S3 has bigger and strongly agglomerated particles, as clearly revealed by the TEM images in Figure 1e and by the reduced surface area compared to S2. The photocurrent density was, however, higher and even very close to that of monodispersed nanoparticles sample S1. This can only be explained by enhanced light scattering in S3.

**Figure 6:**
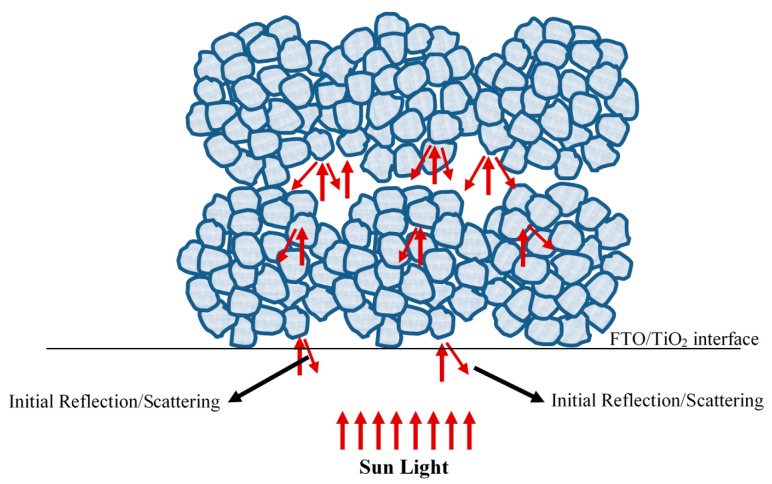
Schematic depicting the expected scattering effects in the DSSC device fabricated using the S3 sample.

Light scattering in DSSCs, successfully explained by the Mie scattering theory [44], is an important process to increase the optical path of solar photons inside the device. This is due to the fact that not all incoming light is absorbed by the nanostructured TiO_2_ film. As mentioned above, a few microns thick scattering layer made of ~300–400 nm TiO_2_ particles is traditionally added on top of the mesoporous nanostructured TiO_2_ layer for this purpose. The S3 sample is fabricated using a single TiO_2_ film composed of the ~200 nm agglomerated spheres, which had a dual function of both dye adsorption and light scattering. Nevertheless, the scattering effect could retrieve 97% of the original photocurrent obtained with the S1 sample. This is an interesting result because of two facts. First, the S3 sample is made of strongly agglomerated spheres that are bigger in size compared to the S2 sample and that have strong inter-agglomeration between the sub-microspheres as well. This definitely results in reduced surface area compared to S2, especially given that both S2 and S3 samples have similar nanocrystallite sizes. Second, a portion of the incident light will initially be reflected at the FTO/TiO_2_ interface (see Figure 6), which is a negative impact of the scattering effect that is not present if a nanoparticle TiO_2_ film is present. The useful effects of light scattering take place only thereafter within the mesoporous layer (see Figure 6) increasing the optical path. Therefore, compared to the S2 sample, S3 has reduced surface area and suffers from initial reflection/scattering at the FTO/TiO_2_ interface. Nonetheless, the scattering within the film itself was efficient enough for S3 to have an enhanced current that is almost equal to the current of the monodispersed sample S1, which has a higher surface area and no expected light scattering. This result illustrates how light scattering can be efficient: despite the initial reflectance and reduced surface area, the scattering effect could retrieve 97% of the original photocurrent. It has to be mentioned that the light scattering effect is not expected to have a significant impact on the light harvesting efficiency for the S2 sample (agglomerate size ~50 nm), as Mie scattering starts to be efficient for scattering particles of sizes only above ~100 nm [45,46,47].

## 4. Conclusions

In conclusion, we report a novel room-temperature solvothermal method to synthesize mesoporous TiO_2_ sub-microspheres that showed strong light scattering effects when implanted as photoanodes in dye-sensitized solar cells. The growth mechanism is explained in terms of non-micelle soft templating of TiO_2_ nanocrystals in a CTAB-based environment. HRTEM and BET surface area measurements confirmed the size and porosity of the prepared samples. When implanted in DSSC devices, the spherical agglomerates of 200 nm size showed strong scattering effects that counter-balanced their reduced surface area and initial reflection at the TiO_2_/FTO interface compared to 15–20 nm monodispersed TiO_2_ nanoparticle films.

## Figures and Tables

**Table 1:** N_2_-isotherm-generated textural properties of prepared samples.

Sample	Surface Area (m^2^/g)	Pore Volume (cm^3^/g)	Pore Size (nm)
S1	40.1	0.29	20
S2	33.9	0.32	28
S3	23.7	0.30	28

**Table 2:** A summary of average extracted *J*-*V* characteristics for dye-sensitized solar cell (DSSC) devices fabricated using S1, S2, and S3 samples.

Sample	*V*_oc_ (V)	*J*_sc_ (mA/cm^2^)	*P*_max_ (mW/cm^2^)	FF	Efficiency (%)
S1	0.672 ± 0.017	13.202 ± 0.392	0.963 ± 0.152	0.575 ± 0.093	5.173 ± 0.870
S2	0.678 ± 0.011	9.987 ± 0.098	0.911 ± 0.019	0.711 ± 0.004	4.912 ± 0.086
S3	0.676 ± 0.003	12.139 ± 0.545	1.023 ± 0.043	0.659 ± 0.004	5.499 ± 0.212
